# Evaluating multimodal large language models for differential diagnosis of high myopia versus high myopia with Glaucoma

**DOI:** 10.3389/fmed.2026.1864498

**Published:** 2026-07-31

**Authors:** Houfa Yin, Tao Yu, Wei Wu, Lixia Shen, Dongmei Shi, Yi Chen, Kaikai Zhao, Andrzej Grzybowski, Kai Jin

**Affiliations:** 1 Eye Center of Second Affiliated Hospital, School of Medicine, Zhejiang University, Hangzhou, China; 2 Zhejiang Provincial Key Laboratory of Ophthalmology, Zhejiang Provincial Clinical Research Center for Eye Diseases, Zhejiang Provincial Engineering Institute on Eye Diseases, Hangzhou, China; 3 School of Clinical Medicine, Hangzhou Medical College, Hangzhou, China; 4 Department of Ophthalmology, University of Warmia and Mazury, Olsztyn, Poland; 5 Institute for Research in Ophthalmology, Foundation for Ophthalmology Development, Poznan, Poland

**Keywords:** chain-of-thought, glaucoma, high myopia, large language model, multimodal diagnosis, ophthalmology, primary open-angle glaucoma

## Abstract

**Background:**

Distinguishing glaucomatous damage from structural and functional changes related to high myopia remains a persistent clinical challenge.

**Objective:**

To evaluate multimodal large language models (LLMs) for the differential diagnosis of high myopia alone versus high myopia with glaucoma under direct-answer and chain-of-thought (CoT) prompting, and to compare physician-rated response quality across models.

**Methods:**

We analyzed a fixed retrospective archive of 100 patients, including 50 patients with high myopia without glaucoma and 50 patients with high myopia and primary open-angle glaucoma (POAG). High myopia was defined according to the source clinical archive as spherical equivalent of −6.0 diopters or less and/or axial length of 26.0 mm or greater. Four multimodal LLMs were assessed in Direct and CoT modes. Two ophthalmologists, each with more than 10 years of clinical experience, independently rated each output on clinical logic, medical knowledge/conclusion accuracy, and evidence use/multimodal consistency; one rater was a chief physician and the other was an attending physician. Diagnostic correctness was compared using Cochran’s Q and McNemar tests; physician ratings were compared using Friedman and Wilcoxon signed-rank tests; weighted Cohen’s kappa and intraclass correlation coefficients (ICCs) were used to assess inter-rater agreement.

**Results:**

In Direct mode, case-level accuracies were 83.0% for Gemini 3 Flash Preview, 75.0% for Kimi-k2.5, 65.0% for GPT-5.4, and 49.0% for Qwen3.5-Plus. In CoT mode, case-level accuracies were 78.0% for Gemini 3 Flash Preview, 75.0% for Qwen3.5-Plus, 73.0% for GPT-5.4, and 69.0% for Kimi-k2.5. Cross-model differences were significant in Direct mode (Cochran’s Q = 31.16, *p* < 0.001) but not in CoT mode (Q = 3.23, *p* = 0.357). Inter-rater agreement was strong, with weighted Cohen’s kappa values of 0.80, 0.84, and 0.83 across the three physician-rated domains and ICC(3, k) = 0.92 for the combined total score.

**Conclusion:**

Multimodal LLMs showed variable performance on this retrospective benchmark for differentiating high myopia from high myopia with glaucoma, but CoT prompting did not consistently improve diagnostic accuracy. Physician ratings provided complementary evidence on clinical coherence and multimodal evidence use, supporting a broader benchmark-oriented evaluation framework for ophthalmic LLM applications.

## 1 Introduction

Glaucoma is a leading cause of irreversible blindness worldwide, and timely diagnosis remains essential for preserving visual function (1). Epidemiologic studies have consistently supported an association between myopia and glaucoma (2, 3). In highly myopic eyes, however, optic disc tilt, peripapillary atrophy, retinal nerve fiber layer thinning, and nonspecific visual field abnormalities can mimic or obscure glaucomatous optic neuropathy (4, 5). Optical coherence tomography (OCT) based structural assessment can still provide useful information in this setting, but conventional parameters may perform unevenly in highly myopic eyes (6–8). Consequently, distinguishing high myopia-related change from true glaucoma remains a persistent challenge in routine clinical practice (9, 10).

Because high-myopia-related and glaucomatous changes can overlap structurally and functionally, this diagnostic problem is inherently multimodal: clinicians usually synthesize optic disc appearance, OCT/RNFL information, visual-field findings, and refractive or axial-length context rather than relying on a single data stream. Conventional artificial intelligence approaches have already achieved strong performance in glaucoma detection from fundus photographs and OCT, supporting the value of image-based computational assessment in ophthalmology (11–14). More recently, large language models (LLMs) and foundation-model paradigms have expanded interest in multimodal clinical reasoning by integrating textual and image-derived information (15–17). In ophthalmology, early LLM studies have examined case-based management questions, patient-facing glaucoma responses, and glaucoma-related prediction tasks (18–20). Other work has focused on education, patient-facing communication, scientific-article interpretation, and broad mapping of the ophthalmic LLM literature (21–23). More recent examples have extended this landscape to glaucoma frequently asked patient questions using both large and locally deployed small language models and to video large language models for quality assessment of dry-eye science popularization videos (24, 25). Nevertheless, these studies largely address information quality, patient-facing education, prediction, or broad ophthalmic LLM mapping rather than the specific multimodal differential-diagnosis problem of distinguishing high myopia alone from high myopia with POAG using fundus photography, OCT, and visual-field materials. Recent reviews have highlighted the rapid expansion of ophthalmic LLM research (26, 27). Parallel reviews of smartphone-enabled artificial intelligence (AI), vision-language foundation models, and broader ophthalmic AI also underscore the need for disease-specific validation under clinically realistic conditions (28–30).

Against this background, the central gap is whether commercial multimodal LLMs can reliably perform this clinically difficult differential diagnosis from multimodal ophthalmic inputs and whether their outputs remain clinically coherent when judged by ophthalmologists. Chain-of-thought (CoT) prompting was evaluated as a secondary prompting strategy because explicit reasoning may make the model’s evidence organization more inspectable, but its reliability in high-stakes diagnostic settings remains uncertain (31). We therefore performed a retrospective benchmark analysis using a fixed 100-case de-identified clinical archive to quantify binary diagnostic accuracy, incorporate blinded physician-rated response-quality assessment, and clarify the implications of Direct versus CoT prompting for this clinically challenging differential-diagnosis task.

## 2 Materials and methods

### 2.1 Study design

**Figure 1:**
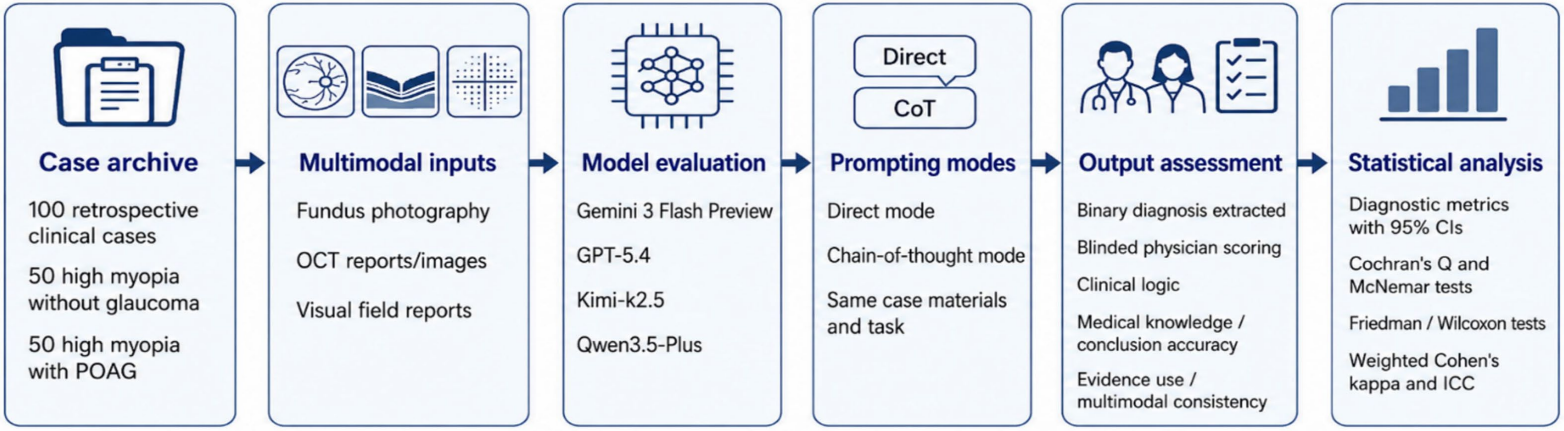
Study workflow showing case assembly, multimodal ophthalmic input preparation, direct and chain-of-thought prompting across four multimodal LLMs, model-output extraction, blinded physician quality scoring, and statistical analysis.

This was a retrospective benchmark analysis of commercial multimodal LLM outputs using a fixed 100-case de-identified single-center clinical archive. The source clinical archive overlaps with a separate related manuscript currently under peer review, entitled “Multimodal Foundation Model Assistance for Differentiating primary open-angle glaucoma (POAG) in Highly Myopic Eyes,” which develops and internally evaluates a RETFound-based multimodal classifier and examines AI-assisted physician reading. The present manuscript addresses a distinct research question: it does not train or evaluate that RETFound classifier, does not assess AI-assisted physician diagnosis, and does not report the reader-assistance endpoints from the related manuscript; instead, it evaluates commercial multimodal LLMs under Direct and CoT prompting and analyzes blinded physician-rated response quality. A case-level master dataset was compiled from model output files and physician scoring workbooks. The overall study workflow is illustrated in Figure 1.

### 2.2 Cases and reference diagnosis

The benchmark comprised 100 archived patients, including 50 patients with high myopia without glaucoma and 50 patients with high myopia and POAG. Because this was an exploratory retrospective benchmark analysis of a fixed single-center archive, no formal *a priori* sample-size calculation was performed; instead, all archived eligible patients with available case materials and reference labels for this LLM benchmark were included, yielding a balanced 100-patient benchmark. In the source clinical archive, high myopia was defined as spherical equivalent of −6.0 diopters or less and/or axial length of 26.0 mm or greater. POAG diagnosis followed the original specialist clinical diagnosis and was based on criteria consistent with the American Academy of Ophthalmology Preferred Practice Pattern for POAG: an open anterior chamber angle together with characteristic glaucomatous optic neuropathy and corresponding functional and/or structural evidence, including visual field defects and/or OCT retinal nerve fiber layer abnormalities (32). Angle-closure glaucoma and secondary glaucoma were not included in this archive. Because this retrospective benchmark used archived clinical labels, the reference standard should be interpreted as specialist clinical diagnosis rather than prospective masked adjudication. Additional subgroup descriptors such as pathologic-myopia status, detailed exclusion categories, and glaucoma severity indices were not uniformly available in the archived files and were therefore not analyzed separately. The reference standard was the expert clinical diagnosis recorded as the ground-truth label in the processed model workbooks.

### 2.3 Inputs and prompting modes

All cases included fundus photographs and OCT reports, and visual-field reports were included for 98–99 of 100 cases across model-specific runs. The multimodal input package did not include clinician-written diagnostic conclusions, free-text diagnostic impressions, or explicit glaucoma/no-glaucoma ground-truth labels. Routine OCT and visual-field reports contained device-generated quantitative indices and normative visualizations, such as retinal nerve fiber layer measurements, visual-field summary indices, probability maps, and color-coded sectors; these report elements were retained as part of standard clinical examination reports and were not separately masked. The case materials, task instruction, and expected response schema were provided in Chinese. The task prompt asked the model to determine whether a highly myopic patient had glaucoma and to return a JSON-formatted response using the Chinese labels “有青光眼” (glaucoma) or “无青光眼” (no glaucoma). All models were evaluated using the same case materials and the same Chinese task instruction; Direct and CoT modes were implemented by disabling or enabling the API reasoning setting, respectively. Detailed prompting instructions and evaluation procedures, including the original Chinese prompt, are provided in the Supplementary methods.

### 2.4 Models

The four evaluated multimodal LLMs were Gemini 3 Flash Preview, GPT-5.4, Kimi-k2.5, and Qwen3.5-Plus. These models were selected to represent contemporary multimodal, API-accessible systems from different major model ecosystems/providers that supported image-plus-text diagnostic prompting at the time of evaluation. Each model was evaluated in Direct and CoT prompting modes using the same case materials and binary diagnostic task.

### 2.5 Inference reproducibility

Diagnostic API calls were made through OpenRouter. The exact API model identifiers recorded in the raw response workbooks were google/gemini-3-flash-preview-20251217 for Gemini 3 Flash Preview, openai/gpt-5.4-20260305 for GPT-5.4, moonshotai/kimi-k2.5–0127 for Kimi-k2.5, and qwen/qwen3.5-plus-20260216 for Qwen3.5-Plus. API calls were completed between March 20 and March 23, 2026. Each case-model-mode combination was submitted once in Direct mode (reasoning.enabled = false) and once in CoT mode (reasoning.enabled = true). The request payloads did not explicitly specify temperature, top_p, max_tokens, or max_completion_tokens. According to OpenRouter API documentation, omitted sampling parameters default to temperature = 1.0 and top_p = 1.0; no user-defined output-token limit was imposed, and output length was governed by the OpenRouter/provider/model limits.

### 2.6 Data governance and API handling

Before API submission, case materials were organized into de-identified multimodal packages. The request payloads included the Chinese task prompt and generic filenames for fundus photographs, OCT reports, and visual-field reports; local source folder names and reference labels were not transmitted. The submitted materials were PNG/PDF exports rather than DICOM objects, and therefore no DICOM tags were submitted. A local audit of the 180 submitted image files found no EXIF metadata, and extractable PDF text and checked PDF metadata fields did not contain source case-folder identifiers. Nevertheless, because PDF technical metadata were not uniformly stripped and the submissions were routed through OpenRouter to commercial model providers, this benchmark should be interpreted as de-identified retrospective research use rather than a fully controlled clinical data-transfer workflow. OpenRouter documentation states that OpenRouter does not store prompts or responses unless users opt in, while downstream providers have their own logging, retention, and training policies. The recorded request payloads did not enable zero-data-retention or provider-specific data-policy routing controls. The study was conducted under institutional ethics approval (No. Y2023-1073); no endpoint-specific data-security clearance document for each commercial API provider was available in the study archive.

### 2.7 Physician scoring

Two ophthalmologists (Ophthalmologist 1 and Ophthalmologist 2) with more than 10 years of clinical experience independently rated every case-model-mode output on three ordinal 1–5 domains: clinical logic, medical knowledge/conclusion accuracy, and evidence use/multimodal consistency. Before scoring, model outputs were anonymized and presented to each ophthalmologist in randomized order. During rating, the ophthalmologists were blinded to model identity, prompting mode, reference diagnosis, and each other’s scores. Ophthalmologist 1 was a chief physician, and Ophthalmologist 2 was an attending physician. A combined physician score was prespecified as the sum of the three domain scores (range, 3–15).

### 2.8 Statistical analysis

Descriptive summaries included case counts, class distribution, and physician score distributions. The primary diagnostic-performance metric was case-level accuracy calculated from the reconstructed case-level dataset. For metric calculation, raw model responses were mapped to binary labels. When automated JSON parsing failed but the raw response contained an explicit “有青光眼” (glaucoma) or “无青光眼” (no glaucoma) diagnosis field, that explicit label was recovered. One Kimi-k2.5 CoT API call produced no content after stopping at the output-length limit; this single failed call was rerun on July 1, 2026 using the same vendor, model identifier (moonshotai/kimi-k2.5–0127), prompt, case materials, and reasoning setting, and the repaired binary output was included in the final metric calculations. No model-mode output remained non-evaluable after this repair. For each model-mode combination, we also calculated sensitivity, specificity, positive predictive value, negative predictive value, and F1 score. Wilson score 95% confidence intervals were calculated for accuracy, sensitivity, specificity, positive predictive value, and negative predictive value. F1-score 95% confidence intervals were estimated by nonparametric bootstrap resampling of case-level predictions with 10,000 replicates. Because binary correctness was paired within cases, Cochran’s Q test was used as the omnibus test for cross-model differences within each prompting mode. When pairwise paired binary comparisons were required, McNemar tests with Holm adjustment were used for cross-model contrasts and for within-model Direct-versus-CoT comparisons. Because physician ratings were ordinal repeated measures, cross-model comparisons used Friedman tests and pairwise Wilcoxon signed-rank tests with Holm adjustment. Weighted Cohen’s kappa quantified dimension-level inter-rater agreement. For the combined total score, ICC(3,k) was prespecified as the primary reliability metric because the benchmark used the average agreement of the two ophthalmologists, whereas ICC(3,1) was reported as a secondary estimate reflecting single-rater reliability.

## 3 Results

The final analytic dataset comprised 100 cases and paired physician evaluations across four evaluated models and two prompting modes.

**Figure 2:**
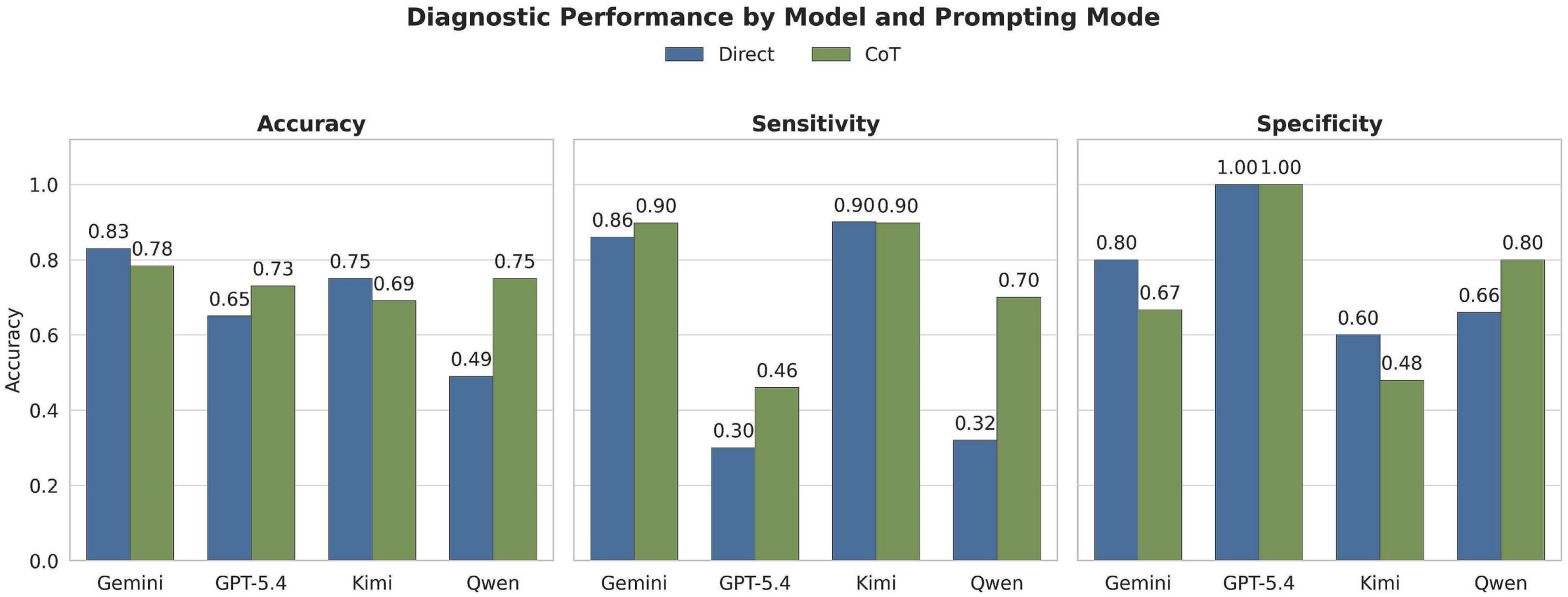
Diagnostic performance of the four evaluated LLMs in direct and chain-of-thought (CoT) modes, showing accuracy, sensitivity, and specificity for each model-mode combination.

Diagnostic performance, including 95% confidence intervals, is summarized in Table 1 and Figure 2. In Direct mode, the highest case-level accuracy was 83.0% for Gemini 3 Flash Preview, followed by 75.0% for Kimi-k2.5, 65.0% for GPT-5.4, and 49.0% for Qwen3.5-Plus. In CoT mode, case-level accuracies ranged from 69.0 to 78.0%. Complete diagnostic performance metrics and interval estimates are provided in Supplementary Table S1.

**Table 1:** Diagnostic performance of each model output by prompting mode.

Model	Mode	Accuracy (95% CI)	Sensitivity (95% CI)	Specificity (95% CI)	PPV (95% CI)	NPV (95% CI)	F1 score (95% CI)
GPT-5.4	CoT	0.730 (0.636–0.807)	0.460 (0.330–0.596)	1.000 (0.929–1.000)	1.000 (0.857–1.000)	0.649 (0.538–0.747)	0.630 (0.486–0.750)
Gemini 3 Flash Preview	CoT	0.780 (0.689–0.850)	0.900 (0.786–0.957)	0.660 (0.522–0.776)	0.726 (0.604–0.821)	0.868 (0.727–0.942)	0.804 (0.712–0.877)
Kimi-k2.5	CoT	0.690 (0.594–0.772)	0.900 (0.786–0.957)	0.480 (0.348–0.615)	0.634 (0.518–0.736)	0.828 (0.655–0.924)	0.744 (0.650–0.825)
Qwen3.5-Plus	CoT	0.750 (0.657–0.825)	0.700 (0.562–0.809)	0.800 (0.670–0.888)	0.778 (0.637–0.875)	0.727 (0.598–0.827)	0.737 (0.627–0.828)
GPT-5.4	Direct	0.650 (0.553–0.736)	0.300 (0.191–0.438)	1.000 (0.929–1.000)	1.000 (0.796–1.000)	0.588 (0.482–0.687)	0.462 (0.302–0.603)
Gemini 3 Flash Preview	Direct	0.830 (0.745–0.891)	0.860 (0.738–0.930)	0.800 (0.670–0.888)	0.811 (0.686–0.894)	0.851 (0.723–0.926)	0.835 (0.747–0.906)
Kimi-k2.5	Direct	0.750 (0.657–0.825)	0.900 (0.786–0.957)	0.600 (0.462–0.724)	0.692 (0.572–0.791)	0.857 (0.706–0.937)	0.783 (0.692–0.859)
Qwen3.5-Plus	Direct	0.490 (0.394–0.587)	0.320 (0.208–0.458)	0.660 (0.522–0.776)	0.485 (0.325–0.648)	0.493 (0.377–0.609)	0.386 (0.247–0.512)

Values are point estimates with 95% confidence intervals.

Omnibus cross-model comparisons of paired binary diagnostic correctness are shown in Table 2. Among the four evaluated models, omnibus differences in Direct-mode correctness were significant by Cochran’s Q test (Q = 31.16, *p* < 0.001), whereas CoT-mode correctness did not differ significantly (Q = 3.23, *p* = 0.357). Within-model Direct-versus-CoT comparisons were significant only for Qwen3.5-Plus after Holm correction (McNemar chi-square = 13.02, adjusted *p* = 0.00138). Pairwise McNemar cross-model comparisons and within-model Direct-versus-CoT comparisons are provided in Supplementary Tables S2, S3.

**Table 2:** Omnibus cross-model comparisons of binary diagnostic correctness by prompting mode.

Mode	Cases	Models	Cochran’s Q	*p* value
Direct	100	4	31.16	<0.001
CoT	100	4	3.23	0.357

**Figure 3:**
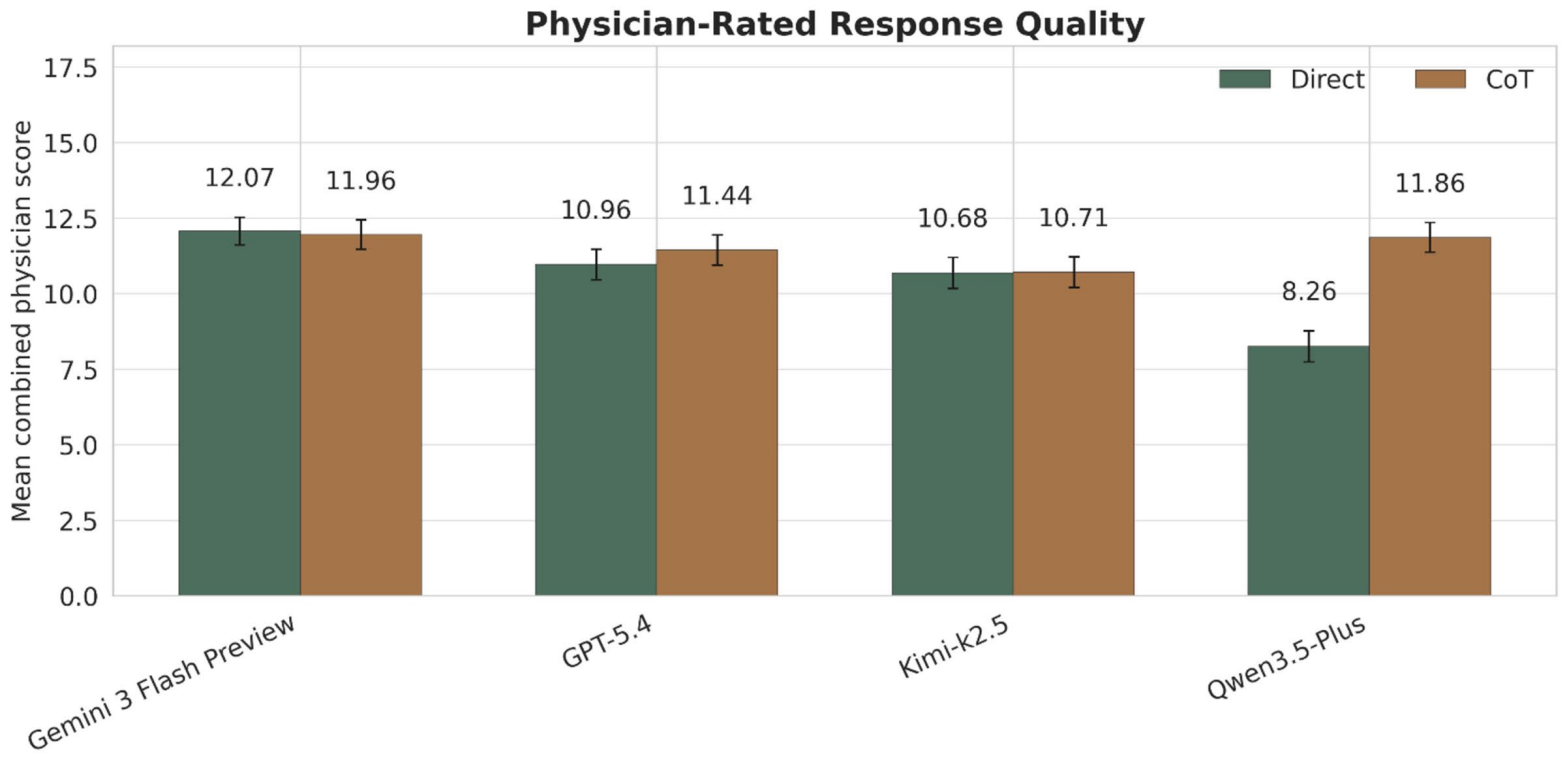
Mean physician-rated combined quality scores for the four evaluated LLMs in Direct and CoT modes. Combined scores represent the sum of ratings for clinical logic, medical knowledge/conclusion accuracy, and evidence use/multimodal consistency.

Physician-rated response quality is summarized in Table 3 and Figure 3. Significant model effects were observed for all three rating dimensions and for the combined physician total score in Direct mode (all *p* < 0.001). In CoT mode, model effects were significant for clinical logic (*p* < 0.001), evidence use/multimodal consistency (*p* = 0.0115), and the combined total score (*p* = 0.00563), but not for medical knowledge/conclusion accuracy (*p* = 0.123). Across both ophthalmologists, Direct-mode outputs from Gemini 3 Flash Preview achieved the highest overall quality scores, whereas CoT-mode outputs narrowed the between-model gap. Omnibus Friedman tests, Holm-significant pairwise Wilcoxon comparisons, and within-model Direct-versus-CoT physician score comparisons are provided in Supplementary Tables S4–S6.

**Table 3:** Physician-rated response quality by model and prompting mode.

Rater	Model	Mode	Clinical logic	Knowledge/conclusion	Evidence use	Combined score
Ophthalmologist 1	GPT-5.4	CoT	3.71	3.75	3.58	11.04
Ophthalmologist 1	GPT-5.4	Direct	3.70	3.70	3.60	11.00
Ophthalmologist 1	Gemini 3 Flash Preview	CoT	3.88	3.80	3.82	11.50
Ophthalmologist 1	Gemini 3 Flash Preview	Direct	3.98	3.91	3.94	11.83
Ophthalmologist 1	Kimi-k2.5	CoT	3.34	3.34	3.22	9.90
Ophthalmologist 1	Kimi-k2.5	Direct	3.45	3.36	3.42	10.23
Ophthalmologist 1	Qwen3.5-Plus	CoT	3.96	3.92	3.88	11.76
Ophthalmologist 1	Qwen3.5-Plus	Direct	2.70	2.70	2.66	8.06
Ophthalmologist 2	GPT-5.4	CoT	3.91	4.01	3.92	11.84
Ophthalmologist 2	GPT-5.4	Direct	3.72	3.71	3.50	10.93
Ophthalmologist 2	Gemini 3 Flash Preview	CoT	4.11	4.22	4.08	12.41
Ophthalmologist 2	Gemini 3 Flash Preview	Direct	4.33	4.02	3.95	12.28
Ophthalmologist 2	Kimi-k2.5	CoT	3.77	3.99	3.76	11.52
Ophthalmologist 2	Kimi-k2.5	Direct	3.92	3.63	3.58	11.13
Ophthalmologist 2	Qwen3.5-Plus	CoT	3.95	4.09	3.94	11.94
Ophthalmologist 2	Qwen3.5-Plus	Direct	3.00	2.91	2.54	8.45

**Figure 4:**
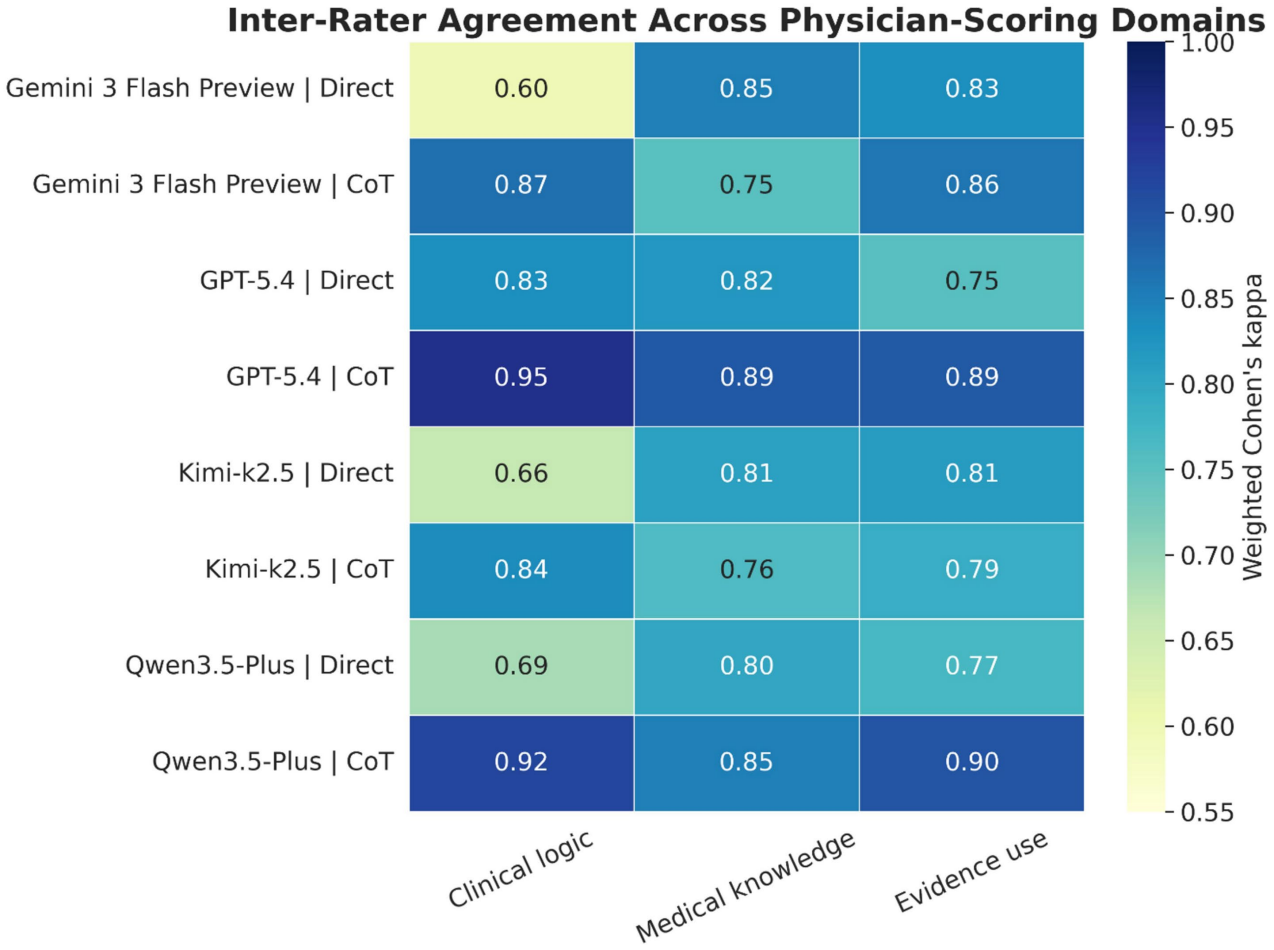
Inter-rater agreement between Ophthalmologist 1 and Ophthalmologist 2 across physician-rating domains, models, and prompting modes. The heatmap summarizes weighted Cohen’s kappa values for clinical logic, medical knowledge/conclusion accuracy, and evidence use/multimodal consistency.

Inter-rater agreement statistics are presented in Table 4 and Figure 4. Across the full physician-scored dataset, weighted Cohen’s kappa values were 0.805 for clinical logic, 0.841 for medical knowledge/conclusion accuracy, and 0.831 for evidence use/multimodal consistency. For the combined total score, ICC(3,1) = 0.859 and ICC(3,k) = 0.924, indicating strong agreement between Ophthalmologist 1 and Ophthalmologist 2.

**Table 4:** Inter-rater agreement across physician scoring dimensions and total score.

Measure	Statistic	Value	Pairs	95% CI
Clinical logic	Weighted Cohen’s kappa	0.805	800	-
Medical knowledge/conclusion accuracy	Weighted Cohen’s kappa	0.841	800	-
Evidence use/multimodal consistency	Weighted Cohen’s kappa	0.831	800	-
Combined total score	ICC(3,1)	0.859	800	[0.840, 0.870]
Combined total score	ICC(3,k)	0.924	800	[0.910, 0.930]

## 4 Discussion

This study extends current ophthalmic LLM evaluation by focusing on a clinically difficult disease pair in which myopia-related structural change can imitate glaucomatous injury. Unlike prior studies centered on case-based management questions or patient-facing glaucoma responses (18, 19), our benchmark required integration of fundus, OCT, and visual field information under explicit differential-diagnosis conditions. Other ophthalmic LLM work has also emphasized educational, communication-oriented, or literature-mapping tasks rather than structurally confounded multimodal diagnosis (21–23). In that sense, the present analysis provides retrospective benchmark evidence for a task in which clinically plausible language is not sufficient and structure–function correlation remains central (5, 9, 10).

The effect of CoT prompting was heterogeneous across models rather than uniformly beneficial. Although explicit reasoning can improve transparency and sometimes performance in general LLM settings (31), recent multimodal CoT studies support a more cautious interpretation for vision-intensive tasks: multimodal CoT can improve VQA-style tasks when rationales are grounded in image features, but generated rationales can also mislead answer inference, and newer benchmarks show that fluent reasoning chains may not be visually grounded or logically coherent and that CoT should be applied selectively across perception and reasoning tasks (33–35). Consistent with this literature, CoT prompting did not provide a stable performance advantage in this retrospective benchmark. Qwen3.5-Plus showed the largest CoT-associated improvement, increasing from 49.0% accuracy in Direct mode to 75.0% in CoT mode, and this was the only within-model comparison that remained significant after Holm correction. In Direct mode, Qwen3.5-Plus predictions were skewed toward the no-glaucoma label (67 no-glaucoma versus 33 glaucoma predictions), with low sensitivity (0.320); under CoT prompting, predictions became more balanced (55 no-glaucoma versus 45 glaucoma predictions), and sensitivity and specificity increased to 0.700 and 0.800, respectively. This pattern is consistent with CoT reducing a Direct-mode response bias or helping the model organize multimodal evidence more consistently, although the single-run design prevents a definitive mechanistic conclusion. In contrast, Gemini 3 Flash Preview decreased from 83.0 to 78.0% under CoT prompting. These divergent patterns indicate that CoT should be interpreted as a model-dependent prompting intervention rather than a generally performance-enhancing strategy.

An important contribution of this study is the addition of physician-rated output quality as a complementary evaluation layer. Binary accuracy alone cannot determine whether a model reached its answer for clinically defensible reasons, whether the explanation remained internally coherent, or whether multimodal evidence was integrated appropriately. This distinction is especially relevant for benchmark studies in ophthalmology, where plausible but weakly grounded explanations could still appear persuasive to clinicians in training or to non-expert users. Consistent with Figure 4, clinical-logic agreement tended to be higher in CoT than in Direct mode across several models, suggesting that explicit reasoning may make response logic more consistently interpretable to physician raters even when CoT does not reliably improve diagnostic correctness. In addition, the combined physician scores clustered within a relatively narrow moderate-to-high range across model-mode combinations, suggesting possible central-tendency effects or restriction of range; this may reduce the sensitivity of the rating scale for separating higher-quality outputs. The strong inter-rater agreement observed here supports the feasibility of combining physician judgment with conventional accuracy metrics in this two-rater benchmark setting; however, ICC and kappa estimates based on only two ophthalmologists should be interpreted cautiously and should not be assumed to generalize to broader multi-rater evaluation settings without further validation.

Our findings should also be interpreted in the broader context of ophthalmic AI development. Earlier deep-learning systems for glaucoma primarily targeted narrower image-classification tasks and often achieved strong performance within specific imaging domains (11–14). More directly related studies have shown that task-specific supervised models can detect glaucoma or glaucomatous optic neuropathy in myopic or highly myopic eyes from OCT or fundus inputs. For example, a multi-task 3D OCT model reported strong glaucomatous optic neuropathy detection with internal AUROC 0.949 and external validation across multiple datasets, a macular vertical OCT model reported AUROC 0.976 for glaucoma detection in myopic eyes with large parapapillary atrophy, and a fundus-photograph study in highly myopic populations reported AUC 0.894, accuracy 82.16%, sensitivity 81.04%, and specificity 83.27% (36–38). A more recent OCT texture-en-face/autoencoder approach in myopic eyes also reported AUROC 0.92 (39). These studies provide important performance context, but they are not direct head-to-head comparators because they used supervised image-classification pipelines, different image formats, different case definitions, and different test sets. The present benchmark instead evaluated commercial multimodal LLMs on mixed fundus, OCT, and visual-field clinical inputs with structured text output and physician-rated reasoning. Therefore, the observed LLM performance should be interpreted as scenario-based benchmark evidence rather than evidence that multimodal LLMs outperform task-specific supervised or foundation-model classifiers.

Several limitations merit emphasis. First, the sample size was modest and derived from a single-center archive, which limits generalizability. Second, the retrospective archive-based design restricted control over potentially relevant covariates and prevented prospective assessment of workflow impact. Third, although all glaucoma cases in the archive were POAG, more granular phenotyping, including pathologic-myopia status, explicit exclusion-pattern annotation, and glaucoma severity measures such as cup-to-disc ratio, visual field stage, or OCT-based damage stage, was not uniformly available for secondary analysis. We therefore could not determine whether model performance differed across mild, moderate, and severe disease. Fourth, this study addressed diagnostic differentiation within a retrospective benchmark rather than downstream management decisions, and the present findings should therefore not be interpreted as evidence of clinical readiness or autonomous deployment. Fifth, each case-model-mode combination was evaluated once using commercial API models; therefore, stochastic sampling variance and possible backend model-version drift could not be quantified. Sixth, although the input files did not contain explicit ground-truth labels or clinician-written diagnostic impressions, routine OCT and visual-field reports retained device-generated quantitative indices and normative visualizations; therefore, the benchmark should be interpreted as an evaluation on real-world multimodal ophthalmic reports rather than on fully annotation-free raw imaging data. Seventh, the commercial API workflow introduces residual data-governance uncertainty: provider-side retention and model-training behavior could not be independently audited retrospectively, PDF technical metadata were not uniformly stripped, and formal endpoint-specific data-security clearance was not documented for each provider. Eighth, we did not train a same-dataset conventional deep-learning or foundation-model comparator. The fixed 100-case benchmark was designed for evaluation of pretrained commercial multimodal LLMs rather than *de novo* supervised training and validation, and this limits direct assessment of whether LLMs offer advantages over optimized supervised image-classification pipelines. Future prospective benchmarks should use institutionally approved API endpoints, provider data-retention controls, a fully documented metadata-stripping pipeline before submission, and prespecified comparisons with task-specific supervised or foundation-model classifiers.

Future work should prioritize externally validated, prospectively assembled multimodal benchmarks with transparent provenance and stronger linkage between model responses and clinically meaningful actions. In particular, future datasets should incorporate prospectively defined glaucoma phenotypes and severity stratification so that model performance can be assessed across earlier and more advanced POAG presentations in highly myopic eyes. Recent reviews have already called for more rigorous and clinically grounded evaluation frameworks for ophthalmic LLMs (26, 27). Broader reviews of ophthalmic AI and foundation-model development similarly suggest that future benchmarks should assess evidence grounding, usability, safety, and reviewer efficiency alongside accuracy (29, 30, 40).

Strengths of this benchmark analysis include paired statistical comparisons, complementary physician-based quality evaluation, and explicit reporting of inter-rater agreement. Limitations include the single-center 100-patient archive, the retrospective design, the absence of prospective validation, incomplete granular phenotyping, partially recoverable request-side inference settings, the limited number of evaluated models, and the uncertainty and limited generalizability of two-rater agreement estimates.

## 5 Conclusion

Multimodal LLMs showed variable performance on this retrospective benchmark for differentiating high myopia from high myopia with POAG. CoT prompting did not consistently improve diagnostic accuracy, whereas physician ratings provided an additional perspective on clinical quality and multimodal evidence integration. Further external validation and prospective benchmarking remain necessary before stronger comparative claims or any clinical-use conclusions can be made.

## Data Availability

The raw data supporting the conclusions of this article will be made available by the authors, without undue reservation.
