# Supplementary material for: Evaluating multimodal large language models for differential diagnosis of high myopia versus high myopia with Glaucoma
Source: Front Med (Lausanne). 2026 Jul 31;13:1864498. doi: 10.3389/fmed.2026.1864498 (PMC13474009; doi:10.3389/fmed.2026.1864498)
Supplement: Supplementary file 1 [file Supplementary_file_1.DOCX]

**Supplementary Material**

*Evaluating Multimodal Large Language Models for Differential Diagnosis of High Myopia Versus High Myopia with Glaucoma*

Houfa Yin^1,2#^, Tao Yu^1,2#^, Wei Wu^1,2^, Lixia Shen^1,2^, Dongmei Shi^1,2^, Yi Chen^3^, Kaikai Zhao^1,2^, Andrzej Grzybowski^4,5^, Kai Jin ^1,2^*

^1^ Eye Center of Second Affiliated Hospital, School of Medicine, Zhejiang University, Hangzhou, China.

^2^ Zhejiang Provincial Key Laboratory of Ophthalmology; Zhejiang Provincial Clinical Research Center for Eye Diseases; Zhejiang Provincial Engineering Institute on Eye Diseases, Hangzhou, China.

^3^ School of Clinical Medicine, Hangzhou Medical College, Hangzhou, China.

^4^ Department of Ophthalmology, University of Warmia and Mazury, Olsztyn, Poland.

^5^ Institute for Research in Ophthalmology, Foundation for Ophthalmology Development, Poznan, Poland.

^＃^ These authors contributed equally to this work and share the first authorship.

*Correspondence: Kai Jin, [jinkai@zju.edu.cn](mailto:jinkai@zju.edu.cn)

This supplementary file provides the numerical results supporting the main manuscript, including diagnostic performance, paired statistical comparisons, and physician-rating analyses.

**Supplementary Methods**

**Prompting protocol and evaluation procedure**

All models were evaluated using the same case materials and the same task instruction. For each case, the multimodal input package included fundus photographs, OCT data, and visual field information. Each model was asked to determine whether the patient had glaucoma in the setting of high myopia. To examine the effect of reasoning style, each case was assessed under two prompting modes. In Direct mode, the model was instructed to provide only a binary diagnostic conclusion. In CoT mode, the model was instructed to provide a brief diagnostic rationale followed by a binary conclusion. Apart from this difference in response style, the task instruction and case materials were kept the same across models and prompting modes.

Standardized task prompts used across models:

Direct prompt:
"Based on the provided multimodal ophthalmic information and relevant medical knowledge, determine whether this highly myopic patient has glaucoma. Please provide only the final diagnosis as either 'glaucoma' or 'no glaucoma'."

CoT prompt:
"Based on the provided multimodal ophthalmic information and relevant medical knowledge, determine whether this highly myopic patient has glaucoma. Please provide a brief diagnostic rationale and then the final diagnosis as either 'glaucoma' or 'no glaucoma'."

The resulting model outputs were recorded for case-level diagnostic accuracy analysis and subsequent physician scoring. This standardized prompting design was intended to keep the clinical task definition consistent across models while isolating the effect of response format on downstream evaluation.

**Physician scoring workflow and masking**

Two ophthalmologists independently scored all model outputs on the three prespecified 1-5 domains reported in the main manuscript.

**Physician scoring rubric**

Scores were assigned on ordinal 1-5 scales for each prespecified domain. To improve rating consistency, anchor definitions were prespecified for scores 1, 3, and 5 in each domain, with scores 2 and 4 used for intermediate performance.

Clinical logic: A score of 5 indicated a complete and well-structured reasoning process, with a clear clinical pathway from observed findings to diagnostic interpretation and final conclusion, with little or no logical discontinuity. A score of 3 indicated generally reasonable reasoning but with noticeable omissions, abbreviated intermediate steps, or partially forced interpretation. A score of 1 indicated disorganized or internally inconsistent reasoning lacking basic clinical logic.

Medical knowledge and conclusion accuracy: A score of 5 indicated accurate glaucoma-related medical knowledge, correct interpretation of key concepts and findings, and a conclusion consistent with the provided case information. A score of 3 indicated partially inaccurate or imprecise knowledge use, although the overall conclusion remained broadly acceptable. A score of 1 indicated serious medical knowledge errors or a clearly inappropriate conclusion that was inconsistent with the clinical facts provided.

Evidence use and multimodal consistency: A score of 5 indicated accurate identification and integration of the key multimodal evidence supporting the conclusion, such as optic disc changes, retinal nerve fiber layer thinning, visual field defects, intraocular pressure findings, angle status, or relevant clinical history. A score of 3 indicated use of some key evidence but with omission of important information or only moderate cross-modal integration. A score of 1 indicated failure to reason from the key evidence or a mismatch between the cited evidence and the stated conclusion.

**Supplementary Tables**

**Table S1. Full diagnostic performance metrics.**

| Model | Mode | Cases | Accuracy | 95% CI low | 95% CI high | Sensitivity | Specificity | PPV | NPV | F1 score |
| --- | --- | --- | --- | --- | --- | --- | --- | --- | --- | --- |
| Gemini 3 Flash Preview | Direct | 100 | 0.830 | 0.745 | 0.891 | 0.860 | 0.800 | 0.811 | 0.851 | 0.835 |
| Gemini 3 Flash Preview | CoT | 100 | 0.78 | 0.692 | 0.854 | 0.898 | 0.667 | 0.733 | 0.865 | 0.807 |
| GPT-5.4 | Direct | 100 | 0.650 | 0.553 | 0.736 | 0.300 | 1.000 | 1.000 | 0.588 | 0.462 |
| GPT-5.4 | CoT | 100 | 0.730 | 0.636 | 0.807 | 0.460 | 1.000 | 1.000 | 0.649 | 0.630 |
| Kimi-k2.5 | Direct | 100 | 0.750 | 0.657 | 0.825 | 0.900 | 0.600 | 0.692 | 0.857 | 0.783 |
| Kimi-k2.5 | CoT | 100 | 0.690 | 0.593 | 0.774 | 0.898 | 0.479 | 0.638 | 0.821 | 0.746 |
| Qwen3.5-Plus | Direct | 100 | 0.490 | 0.394 | 0.587 | 0.320 | 0.660 | 0.485 | 0.493 | 0.386 |
| Qwen3.5-Plus | CoT | 100 | 0.750 | 0.657 | 0.825 | 0.700 | 0.800 | 0.778 | 0.727 | 0.737 |

Table S2. Pairwise McNemar comparisons across the four evaluated models.

| Mode | Model A | Model B | Pairs | A incorrect/B correct | A correct/B incorrect | McNemar statistic | P value | Holm-adjusted p | Holm-significant |
| --- | --- | --- | --- | --- | --- | --- | --- | --- | --- |
| Direct | Gemini 3 Flash Preview | GPT-5.4 | 100 | 10 | 28 | 7.605 | 0.006 | 0.052 | No |
| Direct | Gemini 3 Flash Preview | Kimi-k2.5 | 100 | 6 | 14 | 2.450 | 0.118 | 0.823 | No |
| Direct | Gemini 3 Flash Preview | Qwen3.5-Plus | 100 | 10 | 44 | 20.167 | 7.10e-06 | 8.52e-05 | Yes |
| Direct | GPT-5.4 | Kimi-k2.5 | 100 | 30 | 20 | 1.620 | 0.203 | 1.000 | No |
| Direct | GPT-5.4 | Qwen3.5-Plus | 100 | 5 | 21 | 8.654 | 0.003 | 0.033 | Yes |
| Direct | Kimi-k2.5 | Qwen3.5-Plus | 100 | 17 | 43 | 10.417 | 0.001 | 0.014 | Yes |
| CoT | Gemini 3 Flash Preview | GPT-5.4 | 100 | 18 | 21 | 0.103 | 0.749 | 1.000 | No |
| CoT | Gemini 3 Flash Preview | Kimi-k2.5 | 100 | 4 | 13 | 3.765 | 0.052 | 0.419 | No |
| CoT | Gemini 3 Flash Preview | Qwen3.5-Plus | 100 | 9 | 10 | 0.000 | 1.000 | 1.000 | No |
| CoT | GPT-5.4 | Kimi-k2.5 | 100 | 22 | 28 | 0.500 | 0.480 | 1.000 | No |
| CoT | GPT-5.4 | Qwen3.5-Plus | 100 | 13 | 11 | 0.042 | 0.838 | 1.000 | No |
| CoT | Kimi-k2.5 | Qwen3.5-Plus | 100 | 20 | 12 | 1.531 | 0.216 | 1.000 | No |

Table S3. Within-model Direct-versus-CoT diagnostic comparisons.

| Model | Pairs | Direct incorrect / CoT correct | Direct correct / CoT incorrect | McNemar statistic | P value | Holm-adjusted p | Holm-significant |
| --- | --- | --- | --- | --- | --- | --- | --- |
| Gemini 3 Flash Preview | 100 | 4 | 11 | 2.400 | 0.121 | 0.198 | No |
| GPT-5.4 | 100 | 11 | 3 | 3.500 | 0.061 | 0.184 | No |
| Kimi-k2.5 | 100 | 5 | 13 | 2.722 | 0.099 | 0.198 | No |
| Qwen3.5-Plus | 100 | 37 | 11 | 13.021 | 3.08e-04 | 0.001 | Yes |

Table S4. Omnibus Friedman tests for physician-rated outcomes.

| Mode | Outcome | Cases | Models | Friedman statistic | P value |
| --- | --- | --- | --- | --- | --- |
| Direct | Clinical logic | 100 | 4 | 24.082 | 2.40e-05 |
| Direct | Medical knowledge/conclusion accuracy | 100 | 4 | 26.161 | 8.83e-06 |
| Direct | Evidence use/multimodal consistency | 100 | 4 | 31.101 | 8.10e-07 |
| Direct | Combined total score | 100 | 4 | 27.410 | 4.83e-06 |
| CoT | Clinical logic | 100 | 4 | 17.862 | 4.70e-04 |
| CoT | Medical knowledge/conclusion accuracy | 100 | 4 | 5.770 | 0.123 |
| CoT | Evidence use/multimodal consistency | 100 | 4 | 11.037 | 0.012 |
| CoT | Combined total score | 100 | 4 | 12.582 | 0.006 |

Table S5. Holm-significant pairwise Wilcoxon comparisons for physician scores.

| Mode | Outcome | Model A | Model B | Pairs | Wilcoxon statistic | P value | Effect size r | Median difference | Holm-adjusted p | Holm-significant |
| --- | --- | --- | --- | --- | --- | --- | --- | --- | --- | --- |
| Direct | Clinical logic | GPT-5.4 | Qwen3.5-Plus | 100 | 311 | 2.17e-05 | 0.425 | 0.000 | 9.76e-04 | Yes |
| Direct | Clinical logic | Gemini 3 Flash Preview | Kimi-k2.5 | 100 | 117 | 0.001 | 0.326 | 0.000 | 0.041 | Yes |
| Direct | Clinical logic | Gemini 3 Flash Preview | Qwen3.5-Plus | 100 | 459 | 4.17e-07 | 0.506 | 0.500 | 2.00e-05 | Yes |
| Direct | Clinical logic | Kimi-k2.5 | Qwen3.5-Plus | 100 | 853.5 | 9.10e-04 | 0.332 | 0.000 | 0.034 | Yes |
| Direct | Medical knowledge/conclusion accuracy | GPT-5.4 | Qwen3.5-Plus | 100 | 340 | 3.38e-05 | 0.415 | 0.000 | 0.001 | Yes |
| Direct | Medical knowledge/conclusion accuracy | Gemini 3 Flash Preview | Qwen3.5-Plus | 100 | 517 | 4.92e-05 | 0.406 | 0.500 | 0.002 | Yes |
| Direct | Evidence use/multimodal consistency | GPT-5.4 | Qwen3.5-Plus | 100 | 201.5 | 2.62e-05 | 0.420 | 0.000 | 0.001 | Yes |
| Direct | Evidence use/multimodal consistency | Gemini 3 Flash Preview | Qwen3.5-Plus | 100 | 358.5 | 1.73e-06 | 0.478 | 1.000 | 7.98e-05 | Yes |
| Direct | Evidence use/multimodal consistency | Kimi-k2.5 | Qwen3.5-Plus | 100 | 524 | 0.001 | 0.319 | 0.000 | 0.049 | Yes |
| Direct | Combined total score | GPT-5.4 | Qwen3.5-Plus | 100 | 412.5 | 2.61e-05 | 0.421 | 0.000 | 0.001 | Yes |
| Direct | Combined total score | Gemini 3 Flash Preview | Kimi-k2.5 | 100 | 321 | 7.60e-04 | 0.337 | 0.000 | 0.029 | Yes |
| Direct | Combined total score | Gemini 3 Flash Preview | Qwen3.5-Plus | 100 | 509 | 4.31e-07 | 0.505 | 2.000 | 2.03e-05 | Yes |
| CoT | Clinical logic | Gemini 3 Flash Preview | Kimi-k2.5 | 100 | 85 | 3.73e-04 | 0.356 | 0.000 | 0.015 | Yes |
| CoT | Evidence use/multimodal consistency | Gemini 3 Flash Preview | Kimi-k2.5 | 100 | 72.5 | 0.001 | 0.322 | 0.000 | 0.046 | Yes |
| CoT | Combined total score | Gemini 3 Flash Preview | Kimi-k2.5 | 100 | 196 | 4.46e-04 | 0.351 | 0.000 | 0.017 | Yes |

Table S6. Direct-versus-CoT physician score comparisons.

| Model | Pairs | Wilcoxon statistic | P value | Effect size r | Median difference | Holm-adjusted p | Holm-significant |
| --- | --- | --- | --- | --- | --- | --- | --- |
| Gemini 3 Flash Preview | 100 | 368.5 | 0.057 | 0.190 | 0.000 | 0.172 | No |
| GPT-5.4 | 100 | 676 | 0.905 | 0.012 | 0.000 | 0.905 | No |
| Kimi-k2.5 | 100 | 594 | 0.062 | 0.187 | 0.000 | 0.172 | No |
| Qwen3.5-Plus | 100 | 589.5 | 3.45e-06 | 0.464 | 0.000 | 1.38e-05 | Yes |
